# Multiple ring-enhancing cerebral lesions in systemic lupus erythematosis: a case report

**DOI:** 10.1186/1752-1947-6-172

**Published:** 2012-06-28

**Authors:** Thashi Chang, Chaturaka Rodrigo, Nuwan Ranawaka, Inoshi Atukorala

**Affiliations:** 1 Department of Clinical Medicine, Faculty of Medicine, University of Colombo, Colombo, Sri Lanka; 2 National Hospital of Sri Lanka, University Medical Unit, Colombo 10, Sri Lanka

## Abstract

**Introduction:**

Infectious disease in an immunosuppressed patient is a diagnostic challenge. The clinical presentation and the body’s immune response may be quite different from those seen in an immunocompetent patient with the same infection. It is also a race against time to diagnose, as many of these infections can be fatal without timely intervention.

**Case presentation:**

We present the case of a 39-year-old Sri Lankan woman who was on immunosuppressive treatment for systemic lupus erythematosis and who presented with multiple ring-enhancing lesions of the brain. The most likely diagnosis, given the clinical picture, available investigation results, and characteristics of magnetic resonance imaging, was central nervous system tuberculosis. Owing to the small size of the lesions, a tissue biopsy could not be performed. Our patient responded well to a trial of anti-tuberculosis therapy, and there was clinical and radiological evidence of recovery. A paradoxical reaction with the initiation of anti-tuberculosis therapy was observed and this had to be countered with a prolonged course of steroids.

**Conclusions:**

Our experience and previous evidence from case reports suggest that high-dose steroids for a prolonged period (up to eight weeks) should be administered to counter the initial deterioration after starting anti-tuberculous chemotherapy for central nervous system tuberculomas.

## Introduction

Infectious disease in an immunosuppressed patient presents many challenges. First, the characteristic features of infection seen in an immunocompetent person are absent; instead, there will be a spectrum of unfamiliar and rare manifestations. Second, many atypical organisms that are non-virulent in immunocomptent individuals can cause severe disease. Third, owing to malfunction of the host immune system, diagnostic tests may fail to produce positive results. Fourth, rapid pathogen multiplication and dissemination would afford limited time for diagnosis and treatment to save the life of the patient [1]. To highlight some of these challenges, we report the case of a patient who was on immunosuppressive therapy for systemic lupus erythematosis (SLE) and who developed multiple ring-enhancing lesions throughout the brain.

## Case presentation

A 39-year-old Sri Lankan woman presented with a two-week history of nausea, vomiting, severe headache, progressive drowsiness associated with high-grade intermittent fever, and confusion. SLE had been diagnosed five years before. At that time, she had had signs and symptoms of arthralgia, malar rash, alopecia, and anti-nuclear antibody and anti-double-stranded deoxyribonucleic acid antibody positivity on serum. Hypertension was diagnosed at the same time and was later treated as essential hypertension. The initial presentation of SLE was treated with high-dose prednisolone and mycophenolate mofetil (MMF). The prednisolone dose had been tapered off over the years, but MMF had been continued at high doses (1 g/day). Our patient had been poorly compliant to follow-up and had self-medicated at times with repeat prescriptions of steroids. Although the SLE activity was in remission, she was on inappropriately high doses of immunosuppressants (the indication to start MMF was unclear in the records). Apart from hypertension, her medical history was unremarkable, and she was not on any other immunosuppressants. No relevant sexual history, recent travel history, or contact with animals was reported.

On admission, she was drowsy, febrile, and confused. Her Glasgow Coma Scale score was 12 out of 15. She had no neck stiffness. The results of a cranial nerve examination, including funduscopy, were normal. Bilateral exaggerated tendon reflexes in her limbs and bilateral extensor plantar responses were noted during a neurological examination. She had no lymphadenopathy or peripheral manifestations of active SLE. Her cardiovascular system was normal apart from a blood pressure of 150/90 mmHg. She had a non-tender, firm hepatomegaly extending 3 cm below her costal margin. The results of the rest of the abdominal examination and the respiratory system were normal. Her hemoglobin level was 9.8 mg/dL, her total leukocyte count was 21.5×10^9^/L with 88% neutrophils, her platelet count was 324×10^9^/L, and her blood picture showed a normochromic normocytic anemia with a left shift of leukocytes. Her erythrocyte sedimentation rate was 90 mm/hour, and her C-reactive protein was 46 mg/L (the reference value was less than 6), but her serum complement levels were within normal limits. Her blood and urine cultures were sterile, and her chest X-ray was normal. Transthoracic and transesophageal echocardiograms were normal. An ultrasound scan of her abdomen did not show focal lesions in her liver, which was enlarged, or any other foci of sepsis. Her serum electrolytes, including calcium and magnesium levels, blood glucose, and liver and renal function tests, were also within normal limits.

**Figure 1:**
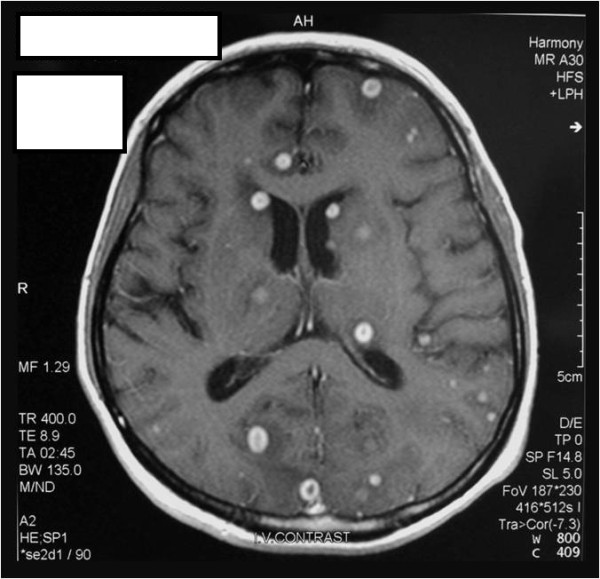
**A magnetic resonance image with gadolinium enhancement at initial presentation.** The image shows multiple ring-enhancing lesions spread diffusely throughout the brain.

Gadolinium-enhanced magnetic resonance imaging (MRI) of her brain showed multiple ring-enhancing lesions that were of varying sizes, up to 1 cm in diameter, and that were surrounded by marked perilesional edema involving her cortex, basal ganglia, brain stem, and cerebellum (Figure 1). There was meningeal enhancement as well.

**Figure 2:**
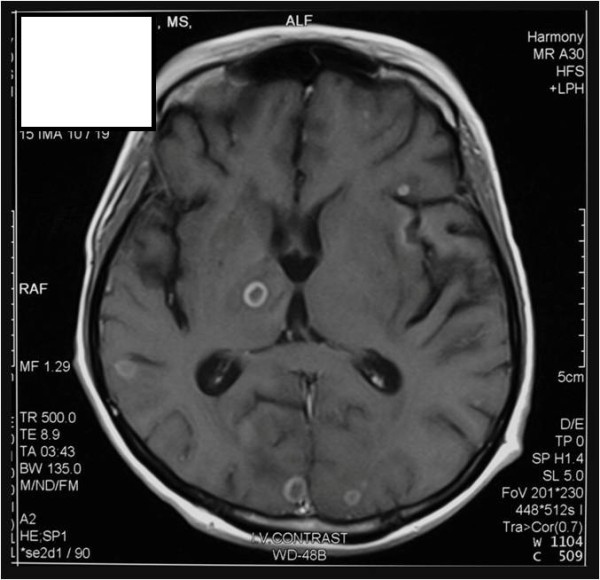
**Magnetic resonance image after three months of anti-tuberculosis therapy.** The image, which corresponds to that of Figure 1, shows a marked reduction in the number of lesions.

A cerebrospinal fluid (CSF) analysis showed lymphocytic pleocytosis, an elevated protein level of 108 mg/dL, and a low CSF-to-serum glucose ratio of 1.8:5.8. There were no atypical cells on CSF cytology, and bacterial, fungal, and tuberculous cultures of CSF were negative. The toxoplasma antibody titers were negative for immunoglobulin M but positive for immunoglobulin G (1:150). The fungal cultures were sterile. The results of the human immunodeficiency virus antibody test were negative. The serum lactate dehydrogenase level was normal. Our patient was initially treated with broad-spectrum intravenous antibiotics with both aerobic and anaerobic cover (meropenem and metronidazole) combined with steroids – dexamethasone 4 mg three times a day (t.i.d.) – to reduce perilesional edema. Owing to the minute size of the lesions, a biopsy for histology was not possible. However, given the poor response to antibiotics and given the likelihood of central nervous system (CNS) tuberculosis (TB) as evidenced by the clinical presentation and context, MRI characteristics, CSF analysis, and elevated inflammatory markers, anti-tuberculous chemotherapy (ATT) was empirically commenced and the steroids (dexamethasone 4 mg t.i.d.) were continued. The treatment doses of ATT were isoniazid 300 mg daily, rifampicin 450 mg daily, ethambutol 800 mg daily, and pyrazinamide 1.5 g daily for the first two months and then isoniazid and rifampicin were continued at the same doses for the rest of the duration. Antibiotics were omitted. The high-grade intermittent fever gradually subsided, but after one month of ATT, our patient became increasingly drowsy with pressure headaches and intractable vomiting, which corresponded to an increase in the size of the brain lesions and perilesional edema seen on MRI (paradoxical reaction). The dose of steroids was increased (dexamethasone 8 mg t.i.d.), and ATT was continued. From the third month of ATT, our patient showed steady improvement in clinical status, including improvement of level of consciousness with resolution of pressure headaches and intractable vomiting, improved appetite, and independent ambulation. A follow-up MRI at the end of the third month showed a significant (approximately 60%) reduction in the number of lesions (Figure 2). The dexamethasone dose was reduced (by reducing both dose and frequency and by titrating dose according to symptoms) and later converted to prednisolone. Our patient was discharged on ATT to be continued for 18 months. At discharge, she was also on 20 mg of prednisolone and 75 mg of azathioprine. On review six months later, she had further improved and was independent in most activities of daily living without any remission of SLE. She was off steroids by this time.

## Discussion

The occurrence of multiple tuberculomas in the brain of immunosuppressed patients has been reported previously, but reports of cerebral tuberculomas that are as extensive as in our patient and that are associated with a good outcome following ATT are rare [2,3]. As differential diagnoses for multiple ring-enhancing cerebral lesions, the following were considered in our patient: tuberculomas, pyogenic abscesses, toxoplasmosis, cysticercosis, fungal infections, and neoplasms such as CNS lymphomas or metastatic deposits.

The background immunosuppression, the insidious onset, MRI characteristics, elevated inflammatory markers, and the lymphocytic pleocytosis in the CSF associated with a high protein and low CSF-to-serum glucose ratio favored TB as the most likely etiology. However, there was no contact history of TB and the chest X-ray was normal. The insidious onset and CSF characteristics were not consistent with pyogenic abscesses, and the results of screening investigations for fungal infections, parasitic infections, and malignancy were negative.

Tuberculomas are conglomerate caseous foci within the substance of the brain and develop from deep-seated tubercles acquired during a recent or remote hematogenous bacillemia. Tuberculomas can involve the brain, spinal cord, and subarachnoid, subdural, or epidural space and may be solitary or multiple. In adults, lesions are found predominantly in the supratentorial compartment in the frontal and parietal lobes. The clinical presentation of tuberculomas is often protean and includes headache, focal neurological signs, seizures, papilloedema, and fever. CSF analysis is usually non-specific, and cultures are often negative. Polymerase chain reaction for tubercle bacilli is insensitive [4]. Therefore, the diagnosis is usually based on neuroimaging characteristics and the clinical context. The radiological presentation depends on whether the granuloma is non-caseating, caseating with a solid center, or caseating with a liquid center. The non-caseating granuloma on contrast-enhanced CT (CECT) is characteristically round, oval, or lobular; varies from 1 to 50 mm; and enhances homogenously. On MRI, these lesions are hyperintense on T2-weighted acquisitions and homogenously enhance with contrast. They are frequently surrounded by a halo of contiguous vasogenic white matter edema [5,6].

In the solid caseating granuloma, the central portion enhances heterogeneously whereas the capsule presents a ring-enhancing pattern. The rim of a caseating TB granuloma is often strikingly hypointense on T2-weighted images and enhances on T1-weighted gadolinium-enhanced MRI. In the next stage, the granuloma with central liquefaction of caseous material is seen as a hypodense core surrounded by a dense ring of enhancement on CECT and T1-weighted gadolinium-enhanced MRI. The activity of a tuberculoma may be judged by the degree of contrast enhancement on follow-up CT or MRI studies.

Multiple cerebral tuberculomas as extensive as those in our patient are rare and are thought to be due to the immunosuppression she was subjected to as part of the treatment for SLE. Such extensive disease is usually associated with a poor prognosis given the enlargement of existing lesions as well as the appearance of new lesions causing increased intracranial pressure [7]. Paradoxical worsening of symptoms following ATT (a previously reported phenomenon termed paradoxical reaction) occurred in our patient but was countered with increased doses of intravenous corticosteroids [8]. In a recent review, it was recommended that intravenous corticosteroids be continued for a longer period (four to eight weeks) to counter the possible initial worsening of clinical status following ATT and to consider histological examination of lesions only if a response was not apparent by eight weeks [2]. Though not directly related to tuberculomas, a meta-analysis by Prasad and Singh [9] also provides evidence that steroid treatment (prednisolone or dexamethasone) reduces mortality (n = 1140 in seven trials, relative risk of 0.78, 95% confidence interval of 0.67 to 0.91) in TB meningitis when combined with ATT.

## Conclusions

Our case report highlights the challenges in the diagnostic workup of immunosuppressed patients presenting with multiple ring-enhancing lesions of the brain, difficulties in establishing a diagnosis of CNS TB, and the paradoxical deterioration following ATT. Our experience and previous evidence from case reports suggest that physicians should not hesitate to continue with high doses of steroids for a prolonged period (up to eight weeks) until the patient recovers from the initial deterioration.

## Consent

Written informed consent was obtained from the patient for publication of this case report and any accompanying images. A copy of the written consent is available for review by the Editor-in-Chief of this journal.

## Abbreviations

ATT, anti-tuberculous chemotherapy; CECT, contrast-enhanced computed tomography; CNS, central nervous system; CSF, cerebrospinal fluid; CT, computed tomography; MMF, mycophenolate mofetil; MRI, magnetic resonance imaging; SLE, systemic lupus erythematosis; TB, tuberculosis; t.i.d., three times a day (ter in die).

## Competing interests

The authors declare that they have no competing interests.

## Authors’ contributions

All authors were involved in the management of the patient. CR and TC did the literature survey and wrote the first draft. All authors read and approved the final manuscript.
